# Molecular dynamics simulation dataset for *x*SiO_2_−*y*B_2_O_3_−(1−*x*−*y*)Na_2_O glasses

**DOI:** 10.1016/j.dib.2024.111218

**Published:** 2024-12-16

**Authors:** Michel Mama Toulou, Paul C.M. Fossati, Cindy L. Rountree

**Affiliations:** a Université Paris-Saclay, CEA, CNRS, SPEC, 91191 Gif-sur-Yvette, France; b Université Paris-Saclay, CEA, Service de Recherche en Corrosion et Comportement des Matériaux, 91190 Gif-sur-Yvette, France

**Keywords:** Oxide glasses, Sodium borosilicate glass, Thermodynamics, Glass structure, Enthalpy of mixing, Elastic properties

## Abstract

Silicate glasses are commonly used for many important industrial applications. As such, the literature provides a wealth of different structural, physical, thermodynamic and mechanical properties for many different chemical compositions of oxide glasses. However, a frequent limitation to existing datasets is that only one or two material properties can be evaluated for a given sample. Another limitation is that existing experimental datasets do not regularly sample a large section of the phase diagram, which makes the determination of systematic trends difficult. Molecular dynamics (MD) simulations are an excellent tool to gather different structural, physical, thermodynamic and mechanical properties on the same glass sample. Additionally, multiple simulations can be setup to homogeneously sample large sections of the phase diagram.

MD simulations were carried out to model glasses of different chemical compositions *x*SiO_2_−*y*B_2_O_3_−(1−*x*−*y*)Na_2_O where *x* and *y* vary from 0 mol% to 100 mol% in 5 mol% increments and *x* + *y* ≥ 50 %. The data presented here includes densities, elementary structural units present in the glass, enthalpies of mixing, elastic moduli, and Poisson's ratio.

To estimate the potential-dependent character of the data, two sets of simulations were run in parallel using two different potentials published by Sundararaman et al. and Wang et al.

The content of this dataset supports interpretations,discussions, and conclusions in the article entitled “Systematic Approach to Thermophysical and Mechanical Properties of SiO_2_–B_2_O_3_–Na_2_O Glasses Using Molecular Dynamics Simulations”.

Specifications Table

**Table:** 

Subject	Computational Materials Science
Specific subject area	Materials properties calculated from Molecular Dynamics (MD) simulations of *x*SiO_2_−*y*B_2_O_3_−(1−*x*−*y*)Na_2_O glasses.
Type of data	Tables (analyzed data in .xslx files)
Data collection	The data were obtained from MD simulations, which were carried out using the LAMMPS code at the TGCC high performance computing center at CEA. The simulations covered a homogenous sampling of the glass-forming composition space of the SiO_2_–B_2_O_3_—Na_2_O pseudo-ternary system. Two different potentials were used to assess the potential-dependent character of the observations. The dataset also includes experimental densities and mechanical properties from then literature, which were used to quantify the validity of both empirical potentials.
Data source location	Université Paris-Saclay, CEA, Service de Recherche en Corrosion et Comportement des Matériaux, 91,190, Gif-sur-Yvette, France
Data accessibility	Repository name: Mendeley Data Data identification number: 10.17632/jm38nkwp9x.1 Direct URL to data: 10.17632/jm38nkwp9x.1 All data can be freely accessed from the URL.
Related research article	M. B. Mama Toulou, P. C. M. Fossati, C. L. Rountree, Systematic Approach to Thermophysical and Mechanical Properties of SiO_2_–B_2_O_3_–Na_2_O Glasses Using Molecular Dynamics Simulations, J. Non-Cryst. Solids 603 (2023), 122,099. 10.1016/j.jnoncrysol.2022.122099

## 1 Value of the Data

• Data found in the current literature are often fragmentary, with each study considering a few select compositions and experimental protocols varying across articles. The present dataset contains fundamental properties for a broad range of compositions spanning the complete glass-forming region of the SiO_2_−B_2_O_3_−Na_2_O pseudo-ternary system [1]. The exact same protocol was used for all the simulations, which makes the data useful to discuss trends and changes in the considered properties as a function of composition.
• Atomic-scale modelers can use this dataset as a benchmark to assess the accuracy of other empirical potentials. This can be done by comparing their prediction to those we obtained from the Sundararaman et al. [2] and Wang et al. [3] potentials, as well as the experimental densities and elastic moduli that are provided in this dataset. Average errors can thus be calculated over the whole composition space, providing metrics for the potentials’ accuracy.
• When particular properties are sought for a glass, the data set provides a *first guess* as to which chemical composition might meet the requirements. This can be done either by considering the closest data points or by local interpolation, due to the good resolution in the composition space. This dataset can also be used in the future for data mining or to develop empirical models to estimate glass properties for arbitrary compositions, in the same way that available models can predict structural features, density, or mechanical properties [4,5].

## 2 Background

Silicate glasses exhibit advantageous properties such as transparency, low thermal expansion, chemical durability, and high electrical resistance, making them suitable for various industrial applications. These properties depend on the chemical composition, which dictates structural, physical, thermodynamic, and mechanical attributes. Systematically understanding this link is crucial to predict a glass' behavior and tailor its characteristics for specific uses.

**Fig. 1:**
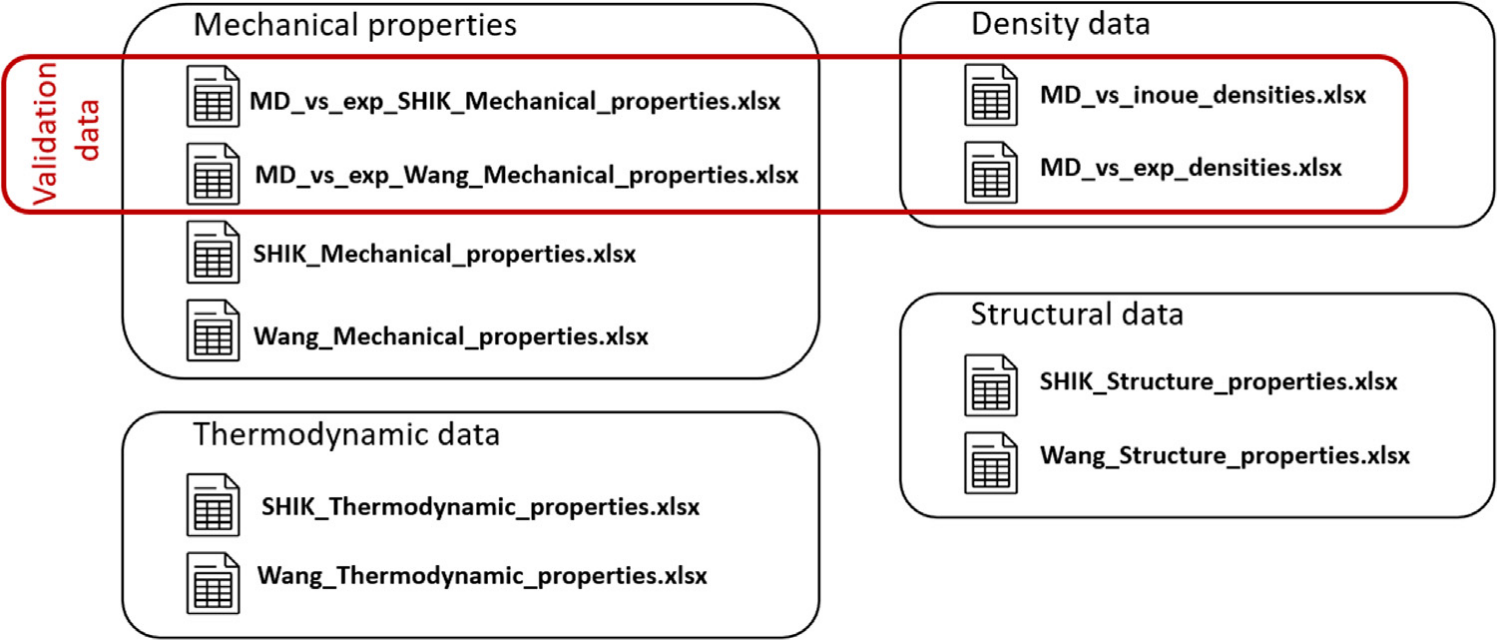
Organization of the files in the dataset.

The three-oxide system composed of SiO_2_, B_2_O_3_, and Na_2_O, known as SBN glasses, is particularly well studied due to its extensive industrial relevance. Despite numerous experimental investigations into the structural, physical, thermodynamic, and mechanical properties of these systems, most studies focus on specific regions of the phase diagram, with limited sampling of the overall composition space. Experimental limitations hinder the acquisition of various properties from a single glass sample, making it challenging to establish systematic trends (Fig. 1).

Molecular dynamics (MD) simulations provide an invaluable tool for overcoming experimental constraints by enabling the simultaneous calculation of multiple properties within the same atomic configuration. The reliability of MD simulations depends on empirical interatomic potentials, which are developed to reproduce selected material properties while predicting others accurately. Several such potentials exist in the literature for SBN glasses, although not all potential sets are equally reliable or versatile. This work employs two distinct potential sets to sample evenly a large part of the SBN oxide glass system's composition space. The notations and abbreviations used in this article are given in Table 1.

**Table 1:** List of symbols and abbreviations used in this article.

Symbol	Units	Meaning
B	GPa	Bulk modulus
G	GPa	Shear modulus
Y	GPa	Young modulus
$R_{\text{SBN}}$	Unitless	Composition ratio $\left[Na_{2}O\right]/\left[B_{2}O_{3}\right]$
$K_{\text{SBN}}$	Unitless	Composition ratio $\left[\text{Si}O_{2}\right]/\left[Na_{2}O\right]$
NN	Unitless	Nearest neighbors
BO	Unitless	Bridging oxygen
NF	Unitless	Network former
NNF	Unitless	Number of nearest neighbors to an oxygen atom, which are network formers
SBN	–	SiO_2_–B_2_O_3_—Na_2_O
$Q^{n}$	Unitless	Silicon tetrahedron with n bridging oxygen ions in its first neighbor shell
$\Phi_{ij}$	eV	Interaction energy between ions i and j
$A_{ij}$	eV	Pre-exponential factor of the Buckingham potentials
$B_{ij}$	Å^-1^	Exponential factor of the Buckingham potentials
$C_{ij}$	eV· Å^6^	Factor for the dispersion interactions
$D_{ij}$	eV· Å^24^	Factor for the short-range interatomic repulsive interactions
$r_{c}$	Å	Cut-off radius
NVT	–	Number of simulated particles (N), simulation cell volume (V) and temperature (T) are constant
NσT	–	number of simulated particles (N), pressure (σ) and temperature (T) are constant
$\Delta H_{\text{mix}}$	eV/atom	Enthalpy of mixing
$\delta \epsilon_{x}$	Unitless	Percent change in the length of the MD simulation box
V	Å^3^	Volume
U	eV	Energy
x	Unitless	Mole fraction of SiO_2_ in the glass
y	Unitless	Mole fraction of B_2_O_3_ in the glass

## 3 Data Description

### 3.1 Density data

The density of glasses is probably one of the most common properties found in literature, as both experiments and simulations easily acquire it. Two files in the Mendeley Data Repository [6] detail the average density for each chemical composition from our calculations.

• **Data file 1: MD_vs_inoue_densities_final.xlsx**: This file compares predictions from our MD calculations with the empirical model by Inoue et al. [5]. The first three columns present the SiO_2_, B_2_O_3_ and Na_2_O concentration in mol% of each oxide in the glass concerning the MD simulations. The next two columns present the average density across the three independent systems acquired using the Wang and SHIK potentials. Inoue et al. [5] provided a method to estimate the density for SiO_2_ −B_2_O_3_ −Na_2_O glasses. Column 6 provides this estimate for each chemical composition. The last two columns concern the relative error between the simulation densities and Inoue et al. [5] values.
• **Data file 2: MD_vs_exp_densities final.xlsx**: This file compares our results with a compilation of experimental measurements from the literature. The first three columns present the SiO_2_, B_2_O_3_ and Na_2_O mol% of each oxide in the glass concerning the MD simulations. The next two columns present the average density across the 3 independent systems acquired using the Wang and SHIK potentials. It is not always possible to find exactly the same chemical composition in literature as in MD simulations; hence, a small tolerance was permitted. Each oxide constitute must be within ±1 mol% of the MD simulation to be considered. Columns 7–8 present a nearest experimental values of SiO_2_, B_2_O_3_ and Na_2_O mol% meeting this criteria, and column 9 presents the density found in literature, with the reference in the subsequent column. At times, more than one author has investigated the same glass; these values have been arbitrarily averaged herein. The last two columns concerns the relative error between simulations and experimental values.

### 3.2 Structural data

The SiO_2_−B_2_O_3_−Na_2_O glasses considered here are well known to be composed of various elementary units (nearest neighbor configurations) which arrange themselves into rings. The elementary units are dictated by the chemical compositions. Data in the Mendeley Data Repository details elementary units concerning the Si, B, and O atoms for each chemical composition averaged over the three independent runs. Structural data are separated into two files: SHIK_Structure_properties_final.xls, which contains properties measured from simulations using the SHIK potential and Wang_Structure_properties_final.xls, which used the Wang potential. Both files have identical formats with the same columns.

• **Data file 3: SHIK_Structure_properties_final.xlsx** and
  **Data file 4: Wang_Structure_properties_final.xlsx**: The first 3 columns present the content of each oxide SiO_2_, B_2_O_3_ and Na_2_O in the glasses, in mol%. The next two (columns 4 and 5) contain the mole ratios $R_{\text{SBN}}$ and $K_{\text{SBN}}$ respectively, which are calculated as
  (1) $$R_{\text{SBN}}=\frac{\left[Na_{2}O\right]}{\left[B_{2}O_{3}\right]}$$
  and
  (2) $$K_{\text{SBN}}=\frac{\left[\text{Si}O_{2}\right]}{\left[B_{2}O_{3}\right]}$$
  Cut-off radii, noted $r_{c}$, were used to determine each atom's nearest neighbors. These cutoffs are different for both silicon (column name: rcSi) and boron (column name: rcB) ions, owing to the difference in the Si–O and B–O bond lengths. They also depend on composition, which also affect bond lengths. They correspond to the minimum in the relevant radial distribution function after the first peak for each simulation. As there are 3 independent simulations per chemical composition, the cutoff radii reported here are the maximum value amongst the different simulations for each composition. Both $r_{c}^{\text{Si}}$ and $r_{c}^{B}$ are presented in the sixth and seventh column, respectively. The eighth and ninth columns show the average coordination of the Si (column name: cSi) and B (column name: cB) atoms. These coordination numbers were calculated by counting the average number of neighbors around both Si and B, according to the cutoff radii defined previously. The subsequent columns present the average number of different structural units across the three independent simulations for each chemical composition. The nomenclature of the columns is as follows:

• The first atom is the atom of interest
• The parenthesis provides information on the structure around the atom of interest
  ○ NN provides the number of nearest neighbors;
  ○ BO provides the number of bridging oxygen atoms amongst those first neighbors;
  ○ NNF is the number of nearest neighbors to an oxygen atom, which are network formers;
  ○ std indicates the standard deviation.

Thus, for example, the number of $Q^{3}$ units is noted Si(NN=4_BO=3), and its standard deviation is Si(NN=4_BO=3)_std. Similarly, the number of bridging oxygen ions is O(NNF=2). Additionally, some structures not traditionally found in SBN glasses occur and are detailed in the table, such as 5-coordinated silicon ions Si(NN=5) or 3-coordinated oxygen ions O(NNF=3). These structures are undoubtedly artefacts resulting from the melt-quench process or the empirical potentials.

### 3.3 Thermodynamic data

MD simulations provide a means to probe thermodynamic properties. Data in the Mendeley Data Repository details various thermodynamic properties. Similarly as for the structural data, there are two files, one per potential:

• **Data file 5: SHIK_Thermodynamic_properties_final.xlsx** and
  **Data file 6: Wang_Thermodynamic_properties_final.xlsx:** The first 5 columns are the same format as for structural measurements: columns 1, 2, and 3 are the SiO_2_, B_2_O_3_ and Na_2_O content in mol%; columns 4 and 5 contain the $R_{\text{SBN}}$ and $K_{\text{SBN}}$ ratios. The following 8 columns detail the average temperature, pressure, volume, total energy, kinetic energy, potential energy, enthalpy and enthalpy of mixing for each chemical composition averaged across the 3 independent simulation boxes. The subsequent columns detail the standard deviation of each of these quantities, in the same order, for each chemical composition averaged across the 3 independent simulation boxes.

### 3.4 Mechanical data

Bulk B, Young's Y and Shear G moduli along with the Poisson's ratio are common mechanical properties estimated from MD simulations. Datasets in the Mendeley Data Repository detail the average of these mechanical properties for each chemical composition along with the standard deviation.

• **Data file 7: SHIK_Mechanical_properties_final.xlsx** and
  **Data file 8: Wang_Mechanical_properties_final.xlsx**: The first 5 columns are the same format as for structural measurements: columns 1, 2, and 3 are the SiO_2_, B_2_O_3_ and Na_2_O content in mol%; columns 4 and 5 contain the $R_{\text{SBN}}$ and $K_{\text{SBN}}$ratios. The columns 6–9 presents the average Bulk B, Young's Y and Shear G moduli and Poisson's ratio ν, respectively. The last 4 columns present the standard deviations concerning the 3 independent simulation runs with the same chemical compositions.

In addition, the dataset also includes files containing mechanical properties from the literature, which were used to assess the quality of the mechanical properties predicted by both empirical potentials:

• **Data file 9: MD_vs_exp_SHIK_Mechanical_properties_final.xlsx** and
  **Data file 10: MD_vs_exp_Wang_Mechanical_properties_final.xlsx:** As stated above, moduli along with the Poisson's ratio are commonly found in literature. Hence, dataset in the Mendeley Data Repository provide a direct comparison with data available in literature. The first 3 columns present the SiO_2_, B_2_O_3_ and Na_2_O content in mol% of each oxide in the glass concerning the MD simulations. The columns 4–7 presents the average Bulk B, Young's Y and Shear G moduli and Poisson's ratio, respectively, generated from MD simulations. It is not always possible to find exactly the same chemical composition in literature as in MD simulations; hence, a small tolerance was permitted. Each oxide constitute must be within ±1 mol% of the MD simulation to be considered. Columns 8–10 present a nearest experimental values of SiO_2_, B_2_O_3_ and Na_2_O mol% meeting this criteria, and columns 11–14 present the Bulk B, Young's Y and Shear G moduli and Poisson's ratio, respectively. Column 15 provides the bibtex key for the reference concerning where the data was found. At times, more that one author has investigated the same glass; these values were arbitrarily averaged herein. The last 4 columns concerns the relative error between simulations and experimental values.

## 4 Experimental Design, Materials and Methods

### 4.1 Model

The content of this dataset results from MD simulations of *x*SiO_2_−*y*B_2_O_3_−(1−*x*−*y*)Na_2_O glasses. We considered glass compositions with the mole fractions *x* and *y* varying 0 to 1 by 0.05 increments. Because sodium-rich glass compositions are mechanically unstable, we considered only the cases where (x+y)≥0.5. We also considered two different empirical potentials that were developed to simulate SBN glasses, developed by Sundararaman et al. [2] (SHIK potential) and by Wang et al. [3]. Both potentials were shown in other works to reproduce thermodynamic properties well for SiO_2_–Na_2_O glasses [7,8]. It should be noted that there is a mistake in the input files used in the original publication of the Wang potential, where the atomic masses were set incorrectly [9]. The input files used in our study were created from the text description in the article by Wang et al. [3], with the correct atomic mass for both boron ions (10.81u) and silicon ions (28.0855u). Both potentials are rigid ions models with fixed charges. For the Wang potential, the ionic charges are independent of the chemical composition, while the oxygen ions’ charge varies depending on composition in the SHIK potential. The non-Coulombic interactions follow the general form

(3) $$\Phi_{ij}\left(r_{ij}\right)=A_{ij}\;e^{-B_{ij}\;r_{ij}\;}-\frac{C_{ij}}{r_{ij}^{6}}+\frac{D_{ij}}{r_{ij}^{24}}$$

where $A_{ij}$, $B_{ij}$, $C_{ij}$ and $D_{ij}$ are empirical parameters adjusted so that the glass simulated with these potentials reproduces various experimental properties. These coefficients are given in Table 2 for both potentials.

**Table 2:** Parameters for the Wang [3] and SHIK [2] potentials.

i–j pair	Wang [3]			SHIK [2]			
	A_ij_	B_ij_	C_ij_	A_ij_	B_ij_	C_ij_	D_ij_
	eV	(Å^–1^)	(eV . Å^6^)	(eV)	(Å^–1^)	(eV . Å^6^)	(eV . Å^24^)
O-O	9022.79	3.774	85.0921	1120.5	2.8927	26.132	16,800
O–Si	50,306.10	6.211	46.2978	23,108	5.0979	139.70	66.0
Si–Si	—	—	—	2798.0	4.4073	0.0	3,423,204
O–Na	120,303.80	5.882	0.0	1,127,566	6.8986	40.562	16,800
Si—Na	—	—	—	495,653	5.4151	0.0	16,800
Na–Na	—	—	—	1476.9	3.4075	0.0	16,800
B–O	206,941.81	8.065	35.0018	16,182	5.6069	59.203	32.0
B–B	484.40	2.857	0.0	1805.5	3.8228	69.174	6000.0
Na–B	—	—	—	3148.5	3.6183	34.000	16,800
B–Si	337.70	3.448	0.0	4798.0	3.6703	207.00	16,800

Electrostatic interactions are calculated using the Wolf summation technique with a convergence factor of 0.2 for the SHIK potential and the particle–particle–particle-mesh (PPPM) method with an accuracy of $10^{-5}$ for the Wang potential. The same cutoff radius of 11Å was used for all short-range interactions, including the Wolf sum for the SHIK potential. The LAMMPS package was used for all MD simulations [10].

### 4.2 Sample creation process

The initial volume of the simulation boxes is chosen for each composition so that the density is 5% lower than predicted by the empirical equations proposed by Inoue et al. [5]. This is done in order to facilitate the initial relaxation by leaving room for the ions to equilibrate. The 12,000 ions are randomly placed in the initial simulation boxes. A minimum interatomic radius of 2Å was enforced in order to avoid pairs of atoms with very short separations. This prevents the very strong attractive interactions when two ions are too close together resulting from the dispersion term of the Buckingham potentials. It also helps limiting the magnitude of the forces at the start of the simulations, which could otherwise result in numerical issues resulting from the use of a finite time step. These simulation boxes then undergo a melt-quench process comprising the following stages:

1. A first high-temperature relaxation at 3000K and fixed volume (NVT conditions). The temperature is controlled using the Tuckerman integrator implemented in LAMMPS, with a characteristic time of 0.1░ps. The purpose of this step is to turn the initial random positions into a realistic high-temperature melt ready for quenching in the subsequent steps. A variable time step is used for the first 10,000 time steps to ensure that the atoms do not move too fast, causing numerical instabilities, which would result in unphysical trajectories.
2. A second high-temperature relaxation of 300░ps at 3,000░K and keeping a fixed volume, during which a snapshot is selected every 100░ps. Thus, there are 3 different relaxed structures for each compositions, which are quenched independently and contribute to the statistical sampling of the different properties.
3. A quench from 3,000░K to 300░K at a cooling rate of $10^{12}\;K\cdot s^{-1}$, using a linear ramping thermostat with the same characteristic time of 0.1░ps.
4. A low-temperature relaxation under ambient conditions of 300░K and 0░GPa (NσT). The shape of the simulation box is allowed to change, subject to a barostat set to 0░GPa. This is to allow the structure to dissipate some of its internal stresses and to reach its natural density. The same thermostat as in the previous step is used, along with a barostat with a damping time of 1░ps.

The resulting structures are used as starting points for the production simulations, over which the relevant properties are calculated. These simulations are done at fixed volume and with the temperature controlled by a Nosé-Hoover thermostat (NVT).

### 4.3 Post-processing

Structural properties such as coordination numbers and the populations of the different structural units depend on of the method used to determine the first coordination sphere of each atom. We use a composition- and potential-dependent criterion for this purpose. This is done by determining the outer size of the coordination shell by locating the minimum after the first peak of the relevant pair distribution functions. These distances are then used to build neighbor lists, which in turn are used to calculate coordination numbers. The neighbor lists are also used to determine the bridging or non-bridging character of each oxygen ion. The combination of the coordination numbers for both network-forming cations and oxygen ions are used to characterize the local environment around each ion, and thus the type of structural unit of which it is part. The cutoff radii used for the different ions pairs are in the data files 3 and 4 for the SHIK and Wang potential, respectively.

The chemical stability of the glasses are indicated by the atomic enthalpy of mixing $\Delta H_{\text{mix}}\left(x,y\right)$, which in turn can be obtained from MD simulation using the expression

(4) $$\begin{array}{c} \Delta H_{\text{mix}}\left(x,\;y\right)=H\left(x,y\right)-x\;H_{\text{Si}O_{2}}-y\;H_{B_{2}O_{3}}-\left(1-x-y\right)H_{Na_{2}O} \end{array}$$

where H(x,y) is the enthalpy of the glass with the composition (x,y), and $H_{\text{Si}O_{2}}$, $H_{B_{2}O_{3}}$, and $H_{Na_{2}O}$ are the end-member enthalpies. The glass enthalpies for each composition were calculated over the production run of all three simulations and then averaged. The reference enthalpies for the end-members were obtained from MD simulations of the end-member compositions after the same melt-quench process as the SBN glasses. The enthalpy of pure Na_2_O has a larger statistical error than the target glasses, due to its tendency to crystallize and the fact that the empirical potentials were not designed for this composition. However, even though this affects the absolute values of the enthalpies of mixing, it does not change the general shape of the enthalpy curves or the qualitative behavior of the glasses.

Beyond structural and thermodynamic properties, MD simulations also provide elastic properties including bulk B, Young Y, and shear G moduli as well as Poisson's ratio ν. The bulk modulus is calculated by applying homogenous deformations to the simulation box, such that its volume is set to V+δV and V−δV before relaxation, with δV=5%. The total energies U are calculated after relaxation, and are then used to calculate the bulk modulus using the finite differences scheme

(5) $$B=V\;\frac{U\left(V+\delta V\right)-2\;U\left(V\right)+U\left(V-\delta V\right)}{\delta V^{2}}$$

The Young modulus is calculated using a similar method as the bulk modulus. In this instance, the box is elongated and contracted along the *x* direction by $\delta \epsilon_{x}\;=\;\pm 0.6\%$. The total energies were calculated after relaxation, and were then used to calculate the Young modulus using the finite differences scheme

(6) $$Y=\frac{1}{V}\;\frac{U\left(\delta \epsilon_{x}\right)-2\;U+U\left(-\delta \epsilon_{x}\right)}{\delta \epsilon_{x}^{2}}$$

## Limitations

Not applicable.

## Ethics Statement

The authors declare that they have read the ethical requirements for publication in Data in Brief. Our work meets all ethical requirements for publication and does not involve human subjects, animal experiments, or any data collected from social media platforms.

## Credit Author Statement

**Michel B. Mama Toulou**: Conceptualization, Methodology, Investigations including running simulations and post-processing, Data Curation; **Paul C. M. Fossati**: Conceptualization, Methodology, Supervision, Writing & Editing, Funding acquisition; **Cindy L. Rountree**: Conceptualization, Supervision, Data Curation, Writing & Editing, Funding acquisition.

## Data Availability

Mendeley DataDataset of sodium borosilicate glass properties from molecular dynamics simulations (Original data).

## References

[1] Mama Toulou M.B., Fossati P.C.M., Rountree C.L. (2023). Systematic approach to thermophysical and mechanical properties of SiO_2_–B_2_O_3_–Na_2_O glasses using molecular dynamics simulations. J. Non Cryst. Solids.

[2] Sundararaman S., Huang L., Ispas S., Kob W. (2020). New interaction potentials for borate glasses with mixed network formers. J. Chem. Phys..

[3] Wang M., Anoop Krishnan N.M., Wang B., Smedskjaer M.M., Mauro J.C., Bauchy M. (2018). A new transferable interatomic potential for molecular dynamics simulations of borosilicate glasses. J. Non Cryst. Solids.

[4] Feil D., Feller S. (1990). The density of sodium borosilicate glasses related to atomic arrangements. J. Non Cryst. Solids.

[5] Inoue H., Masuno A., Watanabe Y., Suzuki K., Iseda T. (2012). Direct calculation of the physical properties of sodium borosilicate glass from its chemical composition using the concept of structural units. J. Am. Ceram. Soc..

[6] Mama Toulou M.B., Fossati P.C.M., Rountree C.L. (2024). Dataset of sodium borosilicate glass properties from molecular dynamics simulations. Mendeley Data.

[7] Fossati P.C.M., Mellan T.A., Kuganathan N., Lee W.E. (2021). Atomistic modeling approach to the thermodynamics of sodium silicate glasses. J. Am. Ceram. Soc..

[8] A. Deshkar, S. Gossé, S. Bégaud-Bordier, P.C.M. Fossati, C.L. Rountree, A multi-scale approach towards thermodynamic assessment of the Na2O-SiO2 phase diagram and thermodynamic properties of sodium silicates, (to be published).

[9] Coudert F.-X. (2023). Failure to reproduce the results of “A new transferable interatomic potential for molecular dynamics simulations of borosilicate glasses. J. Non Cryst. Solids.

[10] Thompson A.P., Aktulga H.M., Berger R., Bolintineanu D.S., Brown W.M., Crozier P.S., In P.J., Veld ’.T., Kohlmeyer A., Moore S.G., Nguyen T.D., Shan R., Stevens M.J., Tranchida J., Trott C., Plimpton S.J. (2022). LAMMPS - a flexible simulation tool for particle-based materials modeling at the atomic, meso, and continuum scales. Comput. Phys. Commun..

